# Technical aspects of quality assurance in radiation oncology

**DOI:** 10.2349/biij.4.3.e48

**Published:** 2008-07-01

**Authors:** CB Saw, MS Ferenci, H Wanger

**Affiliations:** Penn State Hershey Cancer Institute, Hershey, Pennsylvania, United States

**Keywords:** quality assurance, radiation therapy, treatment planning

## Abstract

The technical aspects of quality assurance (QA) in radiation oncology as practice in the United States will be reviewed and updated in the spirit of offering the experience to the radiation oncology communities in the Asia-Pacific region. The word “technical” is used to express the organisational components or processes and not the materials within the QA program. A comprehensive QA program in radiation oncology will have an official statement declaring the quality plan for effective patient care services it provides in a document. The QA program will include all aspects of patient care: physical, clinical, and medical aspects of the services. The document will describe the organisational structure, responsibilities, checks and procedures, and resources allocated to ensure the successful implementation of the quality of patient management. Regulatory guidelines and guidelines from accreditation agencies should be incorporated in the QA program to ensure compliance. The organisational structure will have a multidisciplinary QA committee that has the authority to evaluate continuously the effectiveness of the QA program to provide prompt corrective recommendations and to request feedback as needed to monitor the response. The continuous monitoring aspects require meetings to be held at regular intervals with the minutes of the meetings officially recorded and documented. To ensure that a QA program is effective, the program itself should be audited for quality at regular intervals at least annually. It has been recognised that the current QA program has not kept abreast with the rapid implementation of new and advanced radiation therapy technologies with the most recent in image-based radiation therapy technology. The societal bodies (ASTRO and AAPM) and federal agency (NCI) acknowledge this inadequacy and have held workshops to address this issue. The challenges for the societal bodies and federal agency are numerous that include (a) the prescriptive methodology used may not be appropriate for currently implemented new technologies, (b) resources are becoming scarce, (c) advanced radiation therapy technologies have been introduced too rapidly, (d) advances in radiation therapy technologies have become too sophisticated and specialised with each therapy modality having its own separate set of equipment, for example its own dose planning software, computer system and dose delivery systems requiring individualised QA procedures. At the present time, industrial engineers are being recruited to assist in devising a methodology that is broad-based and more process-oriented risk-based formulation of QA in radiation oncology.

## INTRODUCTION

The rapid deployment of technologically advanced equipment to manage cancer patients has placed stress on the health care systems in the Asia-Pacific region. The incidence of cancers in the Asia-Pacific region has increased significantly over the past several years. This has been attributed to the enormous economic successes with impressive double-digits growth rate per year in the East and Southeast Asia countries and China during the 1980s and 1990s. Such economic successes have led to improved standard of living with better health care systems resulting in fewer deaths from infectious and malnutrition diseases. For example, in the Seventh National Health Development Plan (1992-1996), the Thailand government had increased the health care budget by 54% in five years [1]. As a result, the people in the Asia-Pacific region live longer expanding its population in a much more rapid rate compared to the population in Europe and the USA. Aside from the rapid increase in the general population, the proportion of people who are 65 years old and older has increased at an even more alarming rate. For example, the population of China has increased by 31% over the last 25 years while the people over 65 years old by 81%. By comparison, the population in UK has risen by 6% only and the people older than 65 years by 7% in accordance to the Lancet Asia Medical Forum highlights of the “Asia and Cancer Management in the 21^st^ Century” held in April 21-27, 2007 in Singapore [2]. These disparities are of concerns to the federal agencies of the respective countries in the Asia-Pacific since cancer affects primarily people of older age. If the projection is correct, the number of cancer cases in Asia is set to increase from 3.5 million in 2002 to 8.1 million by 2020 if the current management strategies are not changed [2]. With the change to cancer as a high priority agenda over the previously malnutrition diseases, the federal agencies in their respective countries have to set up new infrastructures to deal with this new disease. Since cancers are the disease of the west, it is natural to import the “know-how” from them to expedite the handling of this disease. This means the importation of technologically advanced equipment, protocols on the training in the proper operation and maintenance of the equipment, and federal guidelines for the safe use of the equipment. This paper has been written with the spirit of offering the experience of the USA on the technical aspects of quality assurance (QA) program in radiation oncology to the radiation oncology communities in the Asia-Pacific region. It will be a review as well as an update engaging in the definition of QA, its organisational structures, the principles underlying QA, and lastly the current state of QA in keeping abreast with the rapid implementation of new image-based radiation therapy technologies. It is the hope of the authors that the presentations will show with sufficient clarity the inter-relationships between the regulatory agencies, the societies of the radiation oncology communities, the hospitals, the radiation oncologists, and its associated staff working synergistically to provide quality of care for patients undergoing radiation therapy.

## HISTORICAL REVIEW

Quality assurance program is well-established and has been integrated into modern radiation oncology practice. This successful integration is attributed, in part to the principal requirement of radiation therapy where the maximum dose delivered is generally limited by normal tissue tolerances and that a large clinical response results from a small change in dose for some tumors. Hence, it is therefore critical that radiation dose must be delivered accurately and consistently. The publication of the International Commission on Radiation Units and Measurements (ICRU) Report No. 24 in 1976 acknowledged that therapeutic systems should be capable of delivering a dose to a volume to within 5% of the prescribed dose [3]. The delivery of radiation therapy in an accurate and consistent manner is by no means simple because of the use of advanced technologically and sophisticated equipment with sets of defined tolerances, the involvement of multi-disciplinary personnel, and the meticulous tasks in the treatment processes. The publication of the American Association of Medical Physicists in Medicine (AAPM) Report No. 13 in 1984 indicated that the possible source of treatment errors include tumor localisation, lack of patient immobilisation, field placement, human errors in calibration, daily patient setup, and equipment-related problems [4]. The subsequent publication in 1994 by AAPM, the Report No. 46 which supersedes AAPM Report No. 13, commonly referred to as the “TG-40 report” presented a comprehensive quality assurance program for radiation oncology practice [5]. While the AAPM Report No. 13 was limited to physical aspects of QA, this later report has expanded into the clinical aspects of patient care addressing those areas which link together the work of the radiation oncologists, radiation therapists, medical dosimetrists (dose planners), and radiation oncology physicists.

The introduction of image-based technologies into radiation oncology practice through three-dimensional (3D) treatment planning systems has transformed the radiation therapy paradigms [6]. Today, conformal radiation therapy (CRT) and intensity-modulated radiation therapy (IMRT) are the standard radiation therapy treatment techniques. These therapy treatment techniques make use of CT-Simulator scanners to acquire patient data making conventional simulators obsolete. Because of the large amount of electronic data acquired, patient data are now downloaded into the treatment planning systems through the electronic data transfer networks. Current treatment planning systems are much more sophisticated in particular having the ability to perform virtual treatment planning and also leaf-sequencing to instruct the dose delivery systems to properly deliver the prescribed dose sequentially. Hence the dose delivery systems with multileaf-collimation capabilities, in some extent, have been automated. In addition, CRT and IMRT require a higher degree of precision and accuracy compared to the traditional treatment techniques. These requirements have led to the introduction of image-guided radiation therapy (IGRT) technology for patient setup and monitoring [7-10]. The ability to monitor patient motion during treatment has lead to the implementation of motion-gated techniques. The radiation oncology community has come to realize that their practice has evolved into a very sophisticated technologically driven radiation therapy practice in a relatively short time. This realisation questions whether the QA program has kept abreast with the advancement of radiation oncology practices [11-12].

## QUALITY ASSURANCE DEFINITION

The term, QA defined by the International Standard Organization (ISO) as “all those planned or systematic actions necessary to provide adequate confidence that a product or service will satisfy given requirements for quality” has been adopted by AAPM TG-40 [5]. In this regard, and as explained in the task group report, the word “quality” refers to the totality of features and characteristics of radiation therapy services that bear on its ability to satisfy the stated or implied goal of effective patient care. The task group elaborated that the QA involves making sure that quality is what it should be. This includes a continuing evaluation of adequacy and effectiveness of quality to ensure prompt implementation of corrective measures and request feedback where necessary. Hence QA can be interpreted simply as a program consisting of systematic checks on various aspects of services to ensure that the declared standards of patient care are maintained. The World Health Organization (WHO) defined QA as all those procedures that ensure consistency of the medical prescription regarding the dose to the target volume, together with minimal dose to normal tissues, minimum exposure to personnel, and adequate patient monitoring to determine the end result of treatment [13].

## COMPREHENSIVE QA PROGRAM

A comprehensive QA program is a quality system built on a quality plan. The quality plan is the official statement that declares the specific quality practices, resources available, and activities related to patient care services it provides. The comprehensive QA program has a document that describes the organisational structure, responsibilities, checks and procedures, and resources allocated to ensure successful implementation of the quality of patient management. This document covers the quality of all aspects of patient care in the radiation therapy processes including (a) personnel qualification, (b) equipment performance, (c) fabrication process, (d) utilisation process, (e) render patient services, and (f) documentation of all aspects of radiation therapy delivery services. In the document, the QA checks, frequency of checks, action criteria, records of checks, and personnel responsible for performing these checks must be clearly defined. The QA program must be sufficiently comprehensive that the cumulative effects of uncertainties associated with complete radiation therapy processes can be determined. This allows tolerance levels and thereafter action criteria to be set for parameters that are being checked. The QA program should have a mechanism of feedback to the QA committee so that any shortcomings can be addressed and resolved promptly. Professional organisations such as the AAPM, American College of Medical Physics (ACMP), and American College of Radiology (ACR) have suggested guidelines in designing the QA program.

The organisational structure should have a Quality Assurance Committee (QAC). The QAC members should consist of radiation oncologist, medical physicist, radiation therapist, nurse, administrator, and personnel as warranted, representing the multidisciplinary nature of the radiation oncology practice. The QAC is charged with authority by the Director of Radiation Oncology and the administration to oversee the QA program. The responsibility of the QAC is to set action levels and delegate QA checks to staff members. For example, the commissioning, proper clinical use, and proper maintenance of all radiation oncology equipment are the responsibilities of a radiation oncologist physicist. The parameter check must be specific such as each member knows his/her responsibilities and be trained to perform checks with the knowledge of what action should be taken if the test or action gives a result outside the tolerance level. Records documenting the frequency and results of every parameter check must be kept in an auditable form for review. The QAC should carefully review instances wherein actions are exceeded, errors are made, and/or procedures are discovered to be faulty, and so on. After the review, the QAC should formulate their recommendations in writing and should retain for audit the minutes of the meeting, the action recommended and the results attained.

A quality audit needs to be performed at regular intervals to assess the need for improvement of the QA program if any. The Joint Commission on Accreditation of Healthcare Organization (JCAHO) requires at least an annual appraisal of the radiation oncology QA program. Although a person within the organisation can perform the quality audit, an outside group should also perform the quality audit as well. In both cases, a person not directly responsible for the areas being audit should perform the quality audit preferably in cooperation with the responsible personnel. Three examples are cited to illustrate the quality audit. The first example is the recommendation of ACR quality assurance program for a monthly audit by a designated reviewer over a sample of patient charts. The second example is the external review of programs, policies, and procedures by ACR. A certificate of compliance will be issued to those facilities that satisfy ACR criteria. Lastly, mailed thermoluminescent dosimeters (TLD) to an external service can be used to verify the treatment unit calibration is consistent with the national standard.

A successful QA program must have dedicated personnel, instruments for measurements, and scheduled time on radiotherapy equipment to perform QA tests. Additional manpower must be allocated to perform QA checks such as daily machine output or audit patient treatment charts. Manpower is also needed to manage the QA program in particular administrative work and coordinating with external organisations in reference to external auditing. QA instruments such as electrometer and ion chamber system are needed to check the performance of the radiotherapy equipment. The availability of time on radiotherapy equipment is of paramount importance because the performance of the equipment is what will be checked. The availability means that time on the treatment machines, simulators, CT-simulators, and treatment planning computer systems must be reserved during normal working hours for QA checks. Such QA checks may include daily constancy measurements performed by the radiation therapist prior to initiating daily treatment, monthly spot check, and annual full calibration on treatment units performed by radiation oncology physicist. There should be continuing education programs to familiarize the QA team members about organisational structure, regulatory guidelines, and updates as adopted in the QA program.

## REGULATORY COMPLIANCE

There are a number of regulations that affect radiation oncology practice. These include the regulations set forth by (a) the state over the operation of radiation producing equipment for medical use, (b) Nuclear Regulatory Commission or agreement state over the medical use of byproduct materials, (c) Environmental Protection Agency (EPA) over the release of materials, (d) Department of Transportation (DOT) over the transport of radioactive materials, (e) Food and Drug Administration (FDA) over the use of radio-pharmaceutical in humans, and (f) FDA and Center for Devices and Radiological Health (CDRH) over the use of medical devices. Any non-compliance to these regulatory guidelines can result in a penalty and an eventual loss of license to practice or operate the equipment. As such, QA program must incorporate compliance with the regulatory guidelines.

Medical centers may seek accreditation from the JCAHO to enhance its quality plan. Although JCAHO accreditation is voluntary, hospitals that do not receive accreditation risk losing medicare reimbursements. JCAHO emphasises the need to support continuous quality improvements (CQI). In addition, JCAHO requires every radiation oncology facility or department to maintain an updated policies and procedure manual. At a minimum, the policy and procedure manual should contain concise description of clinical procedures for patient evaluation, treatment plans, follow-up and morbidity, and mortality reviews. Procedures for treatment planning, treatment delivery, equipment quality assurance, and radiation safety should also be included. The manual should be updated as the procedure change and be reviewed at least once a year. The JCAHO also requires that the radiation oncology program be a part of hospital’s QA program.

## ESTABLISHMENT OF STANDARDS

As stated in AAPM Report No. 13, QA in radiation therapy includes those procedures that ensure a consistent and safe fulfillment of the dose prescription to the target volume with minimal dose to the normal tissues and minimal exposure to personnel. A course of radiation therapy with planning and dose delivery exhibits a continuous process with many feedback loops as explained in ICRU Report No. 24. A system approach to radiation therapy is therefore necessary and QA at the each step is crucial to ensure a proper assessment of the results of the treatment. AAPM Report No. 13 identified radiation therapy as having a clinical component and a physical component. The report only addressed the physical tests and procedures necessary to ensure that a radiation therapy facility can accurately and reproducibly deliver the prescribed dose to the target volume with minimal dose to normal tissue. As declared, the report only addressed the uncertainties associated with dose delivery which will be discussed below.

The AAPM Report No. 13 stated that a QA document needs to have clinically relevant recommendations on acceptable uncertainties in dosimetric procedures and mechanical alignment of the treatment equipment. A large number of parameters, each with its own inaccuracy contributes to the overall uncertainty in the dose delivery yielding a three-dimensional dose distribution to a patient. The uncertainty is determined by separating into random and non-random uncertainties. Random uncertainty is determined using statistical method and be represented by standard deviation. All other uncertainties (non-random uncertainties) are to be estimated in some manner generally as a simple “guesstimate” roughly at one standard deviation of the normal distribution. The non-random uncertainties are combined in quadrature with the random uncertainties to yield a combined uncertainty. The combined uncertainty is multiplied by some factor, 2 or 3 to yield the overall uncertainty that is viewed upon as very approximately to the 95% or 99% confidence interval respectively. This methodology of treating QA allows some flexibility in assigning tolerance values to the individual components of the system. However, the AAPM Report No. 13 alerted that one to realize that (a) the overall impact of all sources of uncertainties must be assessed rather than concentrating on individual component, (b) if the geometric uncertainties predominate, the impact can be different in different areas of the patient, (c) it is necessary to assess the anatomic impact on the treatment uncertainties, and (d) the uncertainties in dose may be non-linearly in relation to the geometric uncertainties and may be asymmetric. With this formalism, tolerance values had been assigned to certain tasks as listed in the AAPM Report No. 13.

## QA OF CLINICAL ASPECTS

The TG-40 report had also expanded QA program addressing the clinical aspects of patient care but excluded the medical aspects such as the decision to treat or the prescription of dose. The emphases of the report are on (a) new patient planning conference, (b) chart reviewing process, (c) chart checking protocol, and (d) film reviewing. The new patient planning conference stresses “the need to know” so that the radiation oncology team can be prepared before the patient arrives at the facility to initiate radiation therapy. During the conference pertinent information about the patient that needs to be presented includes (a) medical history, (b) physical and diagnosis assessment, (c) tumor staging, and (d) treatment strategy. This medical information allows the radiation oncology team to coordinate and schedule the patient in a seamless fashion especially in those cases where the radiation treatment is in combination with chemotherapy or surgery. The condition of the patient at the time of presentation will allow an assessment of whether the patient needs special care in the preparation of immobilisation, whether the patient can undergo treatment, or anticipated changes during treatment. The involvement of medical physics staff is critical at the time of presentation to ensure that the dose plan is designed for the individual patient in accordance to the treatment strategy. Technical issues should be discussed by all participants at the conference to provide appropriate patient positioning and immobilisation to accommodate the patient and delivers the prescribed dose effectively. This include the avoidance in the use of radiation beams that pass through metallic bars of the treatment couch of the machine, the collision of machine components, incomplete coverage of the target due to field size limitation, and/or abutting fields. Also of particular interest to the medical physics staff is the need to know which critical structures should be considered for dose computation at the time of dose planning, then recorded, and thereafter monitored during radiation treatment.

It has been a standard practice to review verification film or portal weekly. However, reviewing portal in a conference attended by the entire treatment team would provide a mechanism of identifying inconsistencies if any between the actual and intended treatment portals by different observers. Once the inconsistency is detected, the cause can be identified and the appropriate action can be discussed and directly communicated. Correction action should be written as directives by the radiation oncologist so that the changes can be implemented as directed. Dose plan and treatment regimen should be discussed to communicate the current approach in regard to the treatment philosophy, i.e., the beam orientation, the number of beams used, or the patient setup. The number of repeated films or portals taken prior to the final portal should be recorded and discussed. The awareness of repeated portals generally indicates difficulty in treatment setup or degradation of the equipment which needs to be investigated and rectified.

## CURRENT STATE OF QA

The introduction of three dimensional (3D) treatment planning systems and innovations in accelerator technology in particular, the introduction of MLC collimation system has transformed the practice of radiation oncology. Patient data is no longer collected manually but extracted from images in particular from CT image dataset. Because of the large amount of digital data, patient data are downloaded into the treatment planning system through the electronic data transfer network. In cases where the tumors are not visible on CT image dataset, multi-modality imaging may be used. The 3D treatment planning systems allow for image fusion and also virtual patient dose planning. After the dose planning, patient machine parameters are downloaded into the database and thereafter retrieved at the machine console also through the electronic data transfer network for dose delivery.

Conformal radiation therapy (CRT) and IMRT treatment techniques are now routinely implemented in the clinics. The stringent requirements of CRT and IMRT for higher level of precision and accuracy of target positioning have led to the introduction of IGRT technology. Forced-breathing and motion-gated technologies have also been introduced to support CRT and IMRT treatment techniques. In the meantime, there have been advances in dose delivery technologies resulting in the introduction of specialised equipment such as the Gammaknife, Cyberknife and Helical Tomotherapy. Each of these therapy machines has its own unique equipment design and hence its own treatment planning software and dose delivery system and thereby requiring individualised QA checks.

Because of the rapid implementation of new and advanced radiation oncology technologies, there have been wide spread concern that current QA practices and protocols do not provide adequately or cost-effectively safeguards against treatment delivery errors that have the potential to degrade the expected therapeutic ratio or in extreme cases, to cause acute injury to the patient undergoing radiation therapy. In September 2005, the National Cancer Institute (NCI) sponsored a quality assurance workshop to address this issue. A follow-up to the workshop was the symposium “Quality Assurance of Radiation Therapy: The Challenges of Advanced Technologies” held in Dallas, Texas on February 20-23, 2007 sponsored jointly by ASTRO, AAPM, and NCI [12]. The first major finding of the symposium is the current process of developing consensus recommendations for prescriptive QA tests remains valid for many of the devices and software systems used in modern radiation therapy, although for some technologies, QA guidance is incomplete or out of date. This finding is understandable with ongoing introduction of new radiation therapy technologies and changes in the principles of QA. The second major finding is the current approach to QA does not seem feasible for image-based planning, image-guided radiation therapy, and/or computer-controlled therapy. These modern technologies rely on electronic data transfer networks, electronic communication software, and software with sophisticated algorithms. The ability to test each individual component would seem overwhelming or impossible. An alternative to testing each component individually is to test the system as a whole. This testing process is often referred to as end-to-end test. End-to-end test has been applied in sophisticated system such as the robotic Cyberknife system used for focal irradiation. The third finding is the need for industrial engineers and human factor experts to make significant contributions towards advancing to a broader, more process-oriented risk-based formulation of radiation therapy QA. Although it sounds logical to have experts in logical systems and risk-based experts to assess the QA in radiation oncology, it is not clear how the recommendations can be implemented. The commitment of resources in terms of manpower or time allocation of machine for QA has been limited. In additional, medical physicists are more involved in the day-to-day clinics then ever taking away time from performing QA. The ability to have more manpower is very dependent on the economics of health care.

## TASK GROUP 100 OF AAPM

The Task Group 100 of AAPM is currently developing a framework in designing quality management (QM) activities that would lead to the allocation of resources based on estimates of clinical outcome, risk assessment, and failure modes [14]. In this framework, a process tree is developed to understand the temporal and physical relationships between the steps involved in the process or procedure, for example an IMRT procedure. The main stream of the process is represented as running along the trunk of the process tree. The tree stems feeding into the tree trunk represent the major processes that are required to execute a successful completion of a process. An analysis methodology referenced to as Failure Mode and Effects Analysis (FMEA) focus on the process and at each step one considers what could possibly goes wrong, how could that happen, and what effects would such a failure produce. There can be several potential failure modes at each step and each failure mode can have several potential causes and outcomes. For example in the IMRT procedure, the potential failure modes can be incorrect patient, corrupted files, or incompatible data in the DICOM data transfer step. The potential causes of the failure are several which could be any, from inadequate training to incompatible DICOM format. The potential effects of the failure could result in the planning on the wrong patient or creating inappropriate dose distribution. For each potential cause of failure, values are assigned in three categories:

- O – the probability that a specific cause will result in a failure mode
- S – the severity of the effects resulting from a specific failure mode should it go undetected throughout the treatment
- D – the probability that the failure mode resulting from the specific cause will go undetected

Conventional values of 1 to 10 are assigned to each category. The product of the three indices yielded the risk probability number (RPN=O*S*D). Decisions are made based on the RPN values. The RPN value of less than 125 is considered unimportant or of little concern.

## CURRENT TREND IN THE INDUSTRY

Today, medical physicists in radiation oncology are more involved in the clinics than ever. This is due in part to (1) the increase use of sophisticated equipment in dose planning and dose delivery and (2) the implementation of clinical procedures requiring expertise from the medical physicists such as prostate implants and patient specific IMRT QA. Because of the transition to image-based technology, medical physicist have also been asked to solve and management networks. Medical physicist are also mandated by regulatory guidelines to be involved in clinical procedures such as treatments using remote afterloading high-dose rate (HDR) brachytherapy systems and the Gamma knife systems. It will be a challenge for medical physicists to allocate additional time for QA with these increased workloads.

With the limited manpower, it is not unusual to have the commissioning of newly purchased medical linear accelerator outsources to an independent vendor. Some medical linear accelerator vendors are also providing experts in commissioning as part of the purchasing package. Medical linear accelerator vendors are now providing the option of having the radiation beam fine tuned to match standard specification of beam referred to as gold beam data. Based on these observations, vendors are aggressively making the posture of providing “ready-to-treat” systems. In the case of interstitial brachytherapy procedures, third party vendors are now available to assay the radioactive seeds, loaded the seeds into needles, and then sterilised them. This third party contract has assisted the medical physicists in performing the clinical procedures of interstitial implants. Based on these trends, it is clear that the radiation oncology community has accepted the fact that medical physicists at the clinics are overwhelmed.

The priority for QA in a radiation oncology facility is expected to be lower. Expecting additional manpower for QA may become more difficult in the future. The expectation of individual institution to perform FMEA for each treatment modality as expected from AAPM TG-100 may be unrealistic. The only logical expectation is for AAPM to provide detailed FMEA but the assignment of values for O, S, and D are somewhat subjective which may not be applicable to all radiation oncology facilities. If the observed trend is as predicted, medical physicists may have time to perform functionality tests only. The evaluation will be based on the end-to-end test methodology. Any assessment will be based on baseline performance and the analysis will be based on a pass/fail test formalism set forth by the vendors. The challenge for future medical physicists will be to identify those critical tasks that must be performed and the appropriate frequency of checks in order to maximize patient care with limited manpower in the individual clinics.

## SUMMARY

The technical aspects of QA program in radiation oncology as practice in the US is described in the spirit of sharing with the radiation oncology communities in the Asia-Pacific region. A comprehensive QA program will have an official statement declaring the quality plan for effective patient care services it provides in a document. The document will also describe the organisational structure, responsibilities, checks and procedures, and resources allocated to ensure the successful implementation of the quality of patient management. Regulatory guidelines and guidelines from accreditation agencies must be included in the QA program to ensure compliance. The organisational structure will have a multidisciplinary QA committee that evaluates the effectiveness of the QA program continuously to provide prompt correctively measures and to request feedback as needed. To ensure that a QA program is effective, the program itself needs to be audited for quality at regular intervals.

**The current QA program has been recognised to be inadequate in keeping pace with the rapid advancement of radiation therapy technologies in particular with the introduction of the image-based technologies. This inadequacy is of concern to the federal agency and societal bodies that led to having workshops to address this issue with the most recent being held in Dallas, Texas in 2007. At the present time, industrial engineers are being recruited to assist in devising a methodology that is broad-based and more process-oriented risk-based formulation of QA in radiation oncology.**
